# New Drugs from the Sea: Pro-Apoptotic Activity of Sponges and Algae Derived Compounds

**DOI:** 10.3390/md17010031

**Published:** 2019-01-07

**Authors:** Giuseppe Ercolano, Paola De Cicco, Angela Ianaro

**Affiliations:** Department of Pharmacy, School of Medicine, University of Naples Federico II, 80131 Naples, Italy; giuseppe.ercolano@unina.it (G.E.); paola.decicco@unina.it (P.D.C.)

**Keywords:** apoptosis, alga, sponge, cancer, marine compound

## Abstract

Natural compounds derived from marine organisms exhibit a wide variety of biological activities. Over the last decades, a great interest has been focused on the anti-tumour role of sponges and algae that constitute the major source of these bioactive metabolites. A substantial number of chemically different structures from different species have demonstrated inhibition of tumour growth and progression by inducing apoptosis in several types of human cancer. The molecular mechanisms by which marine natural products activate apoptosis mainly include (1) a dysregulation of the mitochondrial pathway; (2) the activation of caspases; and/or (3) increase of death signals through transmembrane death receptors. This great variety of mechanisms of action may help to overcome the multitude of resistances exhibited by different tumour specimens. Therefore, products from marine organisms and their synthetic derivates might represent promising sources for new anticancer drugs, both as single agents or as co-adjuvants with other chemotherapeutics. This review will focus on some selected bioactive molecules from sponges and algae with pro-apoptotic potential in tumour cells.

## 1. Introduction

About 71% of the Earth’s surface is water-covered making the marine environment an immense source of new bioactive compounds with uncommon and unique chemical features. Marine organisms, which include about 2.2 million among algae, corals, sponges, and other invertebrates, are highly competitive and complex species, obliged to share a limited extent habitat [1]. The forced coexistence induced the development of exceptional defensive mechanisms to repel or destroy predators, through the production of potent compounds, often referred to as “secondary metabolites”. Those compounds, which are chemically classified as terpenoids, alkaloids, polyketides, peptides, shikimic acid derivatives, sugars, steroids, and a multitude of mixed biogenesis metabolites have been found to exhibit many biological activities (antimicrobial, antitumor, antidiabetic, anticoagulant, antioxidant, anti-inflammatory, antiviral, antimalarial, antitubercular, anti-aging, antifouling, and antiprotozoal) [2]. Among all of these mechanisms, the antitumor activity is certainly the most promising. The first successful marine-derived anticancer drugs was the nucleoside spongothymidine, obtained from the Caribbean sponge *Cryptotethyacrypta* (previously known as *Tethyacrypta*) [3]. In 1969, a derivative of this nucleoside, Ara-C (also known as 1-beta-d-Arabinofuranosylcytosine or cytarabine) was approved by the United States Food and Drug Administration (FDA) under the name of Cytosar-U for the treatment of leukemia and is still in use today for the treatment of acute lymphocytic leukemia (ALL), acute myeloid leukemia (AML), non-Hodgkins lymphoma, and myelodysplastic syndrome (MDS). Nowadays, around 30,000 marine natural products are known, representing a huge industrial and therapeutic potential in virtue of their diversified chemical structures used as privileged scaffolds in drug discovery and development [4]. Overall, more than 3000 new substances identified from marine organisms during the past three decades have shown to have good antitumor activity, and most of them are still in preclinical or early clinical trials, with only a limited number already on the market [5]. To date, five of these compounds have been approved by the FDA for use as pharmaceutical drugs in cancer treatment: Brentuximab Vedotin (Adcetris^®^, for the treatment of patients with Hodgkin lymphoma), Eribulin Mesylate (Halaven^®^, for the treatment of patients with metastatic breast cancer), Trabectedin (Yondelis^®^, for the treatment of patients with unresectable or metastatic liposarcoma or leiomyosarcoma), Fludarabine Phosphate (Fludara^®^, for the treatment of adult patients with B-cell chronic 95 lymphocytic leukemia), and Nelarabine (Arranon^®^, for the treatment of patients with T-cell acute lymphoblastic leukemia and T-cell lymphoblastic lymphoma) (https://www.fda.gov/Drugs/default.htm).

The potential anticancer activity of marine-derived compounds relies on different cellular and molecular mechanisms, including DNA protection, cell-cycle modulation, induction of apoptosis and autophagy, inhibition of angiogenesis, migration, invasion and metastasis formation [6]. Cancer can be considered an extremely heterogeneous disease. Besides, all types of cancer share some characteristics, defined as hallmarks, responsible for their malignant properties [7]. Most of the traditional anti-cancer drugs have been developed to deliberately target one of these hallmarks. However, malignant capabilities of cancer cells are regulated by partially redundant signalling pathways. Consequently, a therapeutic agent inhibiting only one key pathway in a tumour may allow some cancer cells to survive permitting renewed tumour growth and clinical relapse. Thus, there is actually great attention in exploiting strategies able to simultaneously target these supporting pathways, thus preventing the development of adaptive resistance [8]. Natural compounds usually inhibit cancer formation and development through the interaction with multiple cellular signalling pathways. In addition, they are often characterized by a better safety profile than traditional chemotherapy agents, and they are reasonably priced and easily available [9].

Impairment or resistance to apoptosis has been considered probably the most important challenge in oncology. The molecular mechanisms by which tumour cells gain resistance to apoptosis mainly include (1) a dysregulation of the mitochondrial pathway; (2) the inactivation or the loss of caspases; and/or (3) a deficiency of death signals through the transmembrane death receptors of the tumour necrosis factor (TNF) superfamily [10]. Compounds directly inducing apoptosis, reduce the appearance of drug resistance, decrease mutagenesis and reduce toxicity. In the last decade, numerous apoptosis-inducer compounds have been isolated from marine sponges and algae. This review will focus on some selected agents of marine source, which have been reported to affect apoptotic pathways in tumour cells. Some of them with the relative IC_50_ and the chemical structure are shown in Table 1.

## 2. Apoptosis: A Target for Anticancer Therapy

Resistance to apoptosis is one of the hallmarks of human cancers and has been reported to be involved in cancer cells evasion to immune-mediated and cytotoxic drug treatment [11].

The expression “apoptosis” was minted by Kerr et al. [12] in 1970 to describe a specific morphological aspect of cell death. It is a highly regulated process that occurs normally during development and aging and acts as a homeostatic mechanism to remove any needless or undesired cells. Apoptosis also takes place as a defence mechanism such as in immune reactions or when cells are flawed by diseases or toxic compounds [13].

There are two main apoptotic pathways: the extrinsic or death receptor pathway and the intrinsic or mitochondrial pathway that are activated by both intracellular and extracellular signals. The intracellular signals include ultraviolet radiations, DNA damage and growth factor and cytokine deprivation, whereas the extracellular signals involve death receptors (DR) that are members of the TNF receptor gene superfamily and play a critical role in transmitting the death signal from the cell surface to the intracellular signalling pathways [14]. The best-characterized ligands and corresponding death receptors include FasL/FasR, TNF-α/TNFR1, Apo3L/DR3, Apo2L/DR4 and Apo2L/DR5 [15]. However, both extrinsic and intrinsic pathways are linked and converge at the final phase of apoptosis with the activation of the executive cysteine aspartyl-specific proteases (caspases). Caspases are a class of cysteine proteases that cleave target proteins which coordinate the apoptotic process. Caspases are broadly expressed in their inactivated form (named pro-caspases) in most cells and once activated can in turn activate other pro-caspases, amplifying the apoptotic signalling pathway and leading to rapid cell death. There are more than 13 known caspases that are classified into: initiators, effectors, inflammatory and other caspases. In particular, the most important caspases identified and widely studied are caspase-2, -8, -9, -10 as initiators and caspase-3, -6, -7 as executioners [16].

### 2.1. Extrinsic or Death Receptor Pathway

The extrinsic pathway is best represented by the FasL/FasR and TNF-α/TNFR1 models. After the binding of the ligand (FasL or TNF-α) to the respective receptor (FasR or TNFR1) an adaptor protein binds to the complex ligand/receptor. These proteins are namely Fas-associated death domain (FADD) and TNF receptor-associated death domain (TRADD) [17]. Adaptor proteins allow the binding and consequent activation of procaspase-8 and -10 in order to form the death-inducing signalling complex (DISC) [18]. Once caspases-8 and -10 are activated the execution phase of apoptosis starts by involving the effector caspases-3, -6 and -7. The extrinsic pathway can be inhibited by cellular FLICE (FADD-like IL-1β-converting enzyme)-inhibitory protein (c-FLIP) through the interaction with DR5, FADD and caspase-8 which results in an apoptotic inhibitory complex (AIC). In fact, increased expression of c-FLIP has been reported in different types of cancer [19]. Another extrinsic pathway inhibitor protein is toso, a member of the immunoglobulin gene superfamily, that inhibits TNF-α and Fas signalling pathways and has been identified in several human cancer cell lines [20].

### 2.2. Intrinsic or Mitochondrial Pathway

The intrinsic signalling pathway is initiated by different stimuli such as toxins, hypoxia and infections. These stimuli change the mitochondrial permeability inducing the release of different pro-apoptotic proteins into the citosol. The most important mitochondrial proteins are: cytochrome c, apoptosis-inducing factor (AIF) and caspase-activated DNase (CAD) [21]. Upon the release, cytochrome c binds to the apoptotic protease activating factor-1(APAF-1) and procaspase-9, forming a complex named apoptosome in which pro-caspase-9 is converted into caspase-9. Once activated, caspase-9 triggers the effector caspases-3 and -7 promptly leading to cell death [22]. Apoptosis-inducing factor and CAD migrate into the nucleus during the late phase of apoptosis causing DNA fragmentation. Apoptosis-inducing factor works in a caspase independent manner, whereas CAD requires caspase-3 binding for inducing DNA fragmentation [23]. All the intrinsic pathway events are regulated by B-cell lymphoma-2 (BCL-2) protein family that can exert both pro- or anti-apoptotic effects. Some of the anti-apoptotic proteins comprise Bcl-2, Bcl-x and BAG, and some of the pro-apoptotic proteins include Bcl-10, Bax, Bak, Bad and Blk. Overexpression or loss of pro- and anti-apoptotic BCL-2 proteins, respectively, is typical in a wide range of human cancer and has frequently been linked with increased chemoresistance and radioresistance [24].

### 2.3. Other Atypical Forms of Cell Death

Although the use of the term “programmed cell death” is specifically ascribed to apoptosis, there are other different types of cell death which have been evaluated in cancer treatment.

*Mitotic catastrophe* is a cell death mode that occurs during the metaphase in case of dysregulated mitosis and is characterized by the activation of caspase-2 [25]. Mitotic catastrophe was suggested to be an onco-suppressive mechanism. Thus, faltered mitotic catastrophe can promote the uncontrollable growth of cancer cells [26].

*Anoikis* is a particular type of apoptosis induced by cell detachment from extracellular matrix having an important role in preventing anchorage-independent growth and epithelial–mesenchymal transition which are typical features of metastasis development. Cancer cells develop anoikis resistance due to several mechanisms, including alteration of integrin, β-catenin/TCF and other pathways involved in cancer development and progression [27].

*Pyroptosis* is an inflammation related cell death activated by a wide range of stimuli [28]. Pyroptosis is triggered by caspase-1 which is activated by inflammasome, and it is responsible for the production of interleukin 1-beta (IL1-β) and IL-18. Recently, it has been hypothesized that pyroptosis could be exploited as a new and prominent target to mediate anti-cancer treatments [29]. In addition, there are other different types of atypical forms of cell death including: excitotoxicity, Wallerian degeneration, paraptosis, pyronecrosis and entosis [30].

## 3. Marine-Derived Compounds: Inductors of Apoptosis

### 3.1. Marine Sponges

Marine sponges are multicellular aquatic animals that filter water through their porous eating bacteria and other particles. There are more than 5000 various species of marine sponges rich in different essential components such as fatty acids, proteins, alkaloids, peroxides and terpenes showing antibacterial, antiviral, antifungal, anti-malarial, anti-helminthic, immune-suppressive and anti-inflammatory effects [31]. In addition, at least 60 different species of sponges possess also chemopreventive and anticancer effects [32]. Unfortunately, their possible use as anticancer drugs is difficult because of the limited supply of the compounds, which are present only in very few amounts in the sponges. However, in the last few years, the synthetic and/or semi-synthetic chemistry applications for natural products have been reported to be a great starting points for generating such compounds [33]. A list of marine spongean compounds with pro-apoptotic effects is reported in Table 2. 

#### 3.1.1. Alkaloids Isolated from Marine Sponges

Aaptaminoids are alkaloids isolated from different species of *Aaptos* sponge such as aaptamine, demethylaaptamine, isoaaptamine, aaptosamine, aaptosine and demethyloxy-aaptamine [34]. These alkaloids have been reported to possess different biological activity including anti-neoplastic effect, as demonstrated on murine lymphocytic leukemia P-338 cells, human mouth epidermoid carcinoma KB16 cells, human lung adenocarcinoma A549 and human colon adenocarcinoma HT-29 cells [35]. In particular, aaptamine exhibits DNA intercalating activity and induces G2/M cell cycle arrest of human chronic myeloid leukemia K562 cells [36]. In 2014, Dyshlovoy et al. [37] reported that aaptamine at high but non-toxic concentration induces AP-1 and NF-*κ*B- dependent transcriptional activity which are involved in the induction of apoptosis and in cell transformation. Isoaaptamine was also demonstrated to exert cell growth suppression and apoptosis induction in human breast cancer T-47D cells confirmed by the activation of caspase-3 and -7 and the cleavage of their substrate Poly (ADP-ribose) polymerase (PARP) [38].

Crambescidin 800 is a guanidine alkaloid extracted from sponges of genus *Monanchora*. It induces differentiation of K562 cells into erythroblasts and accumulation of cells in the S-phase [39]. However, its anti-proliferative and pro-apoptotic effect has been recently demonstrated in triple negative breast cancer cells T11 and SUM159PT and further confirmed by the cleavage of caspase-3 [40].

Fascaplysin is an alkaloid isolated from the Fijian sponge *Fascaplysinopsis* that exhibits a wide range of pharmacological activities such as antibacterial, antiviral, antifungal and antimalarial. In 2015 it was reported for the first time that fascaplysin induces caspases-mediated apoptosis in HL-60 human pro-myelocytic leukemia cells. In fact, treatment of HL-60 with fascaplysin in the range of nanomolar concentration is able to induce apoptosis in HL-60 cells. In addition fascaplysin also induces autophagy in HL-60 cells and attenuates PI3K/AKT/mTOR signalling [41]. More recently, Rath et al. [42] demonstrated that fascaplysin exerts an anti-proliferative and pro-apoptotic effect in lung cancer and small-cell lung cancer circulating tumour cell lines by involving AKT/PKB and adenosine monophosphate-activated protein kinase (AMPK) pathways.

Lamellarins are a class of alkaloids extracted from different organisms, including molluscs, ascidians and sponges. Lamellarin D represents the lead compound and is the most widely studied in cancer contexts. It showed a potent cytotoxic and pro-apoptotic effect in both human and mouse leukemia cells (Jurkat and P388, respectively). The pro-apoptotic effect of lamellar D is strictly correlated to a direct effect on mitochondria. In fact, lamellarin D induces mitochondrial depolarization which is correlated with nuclear apoptosis [43]. This data has been further confirmed by the addition of cyclosporin A, a potent inhibitor of mitochondrial permeability transition, that totally reverted the pro-apoptotic effect of lamellarin D [44].

Makaluvamines are a class of alkaloids isolated from the marine sponge of genus *Zyzzya*. They showed promising cytotoxic activity in colon cancer cells HCT 116 [45]. Given their interesting activity in cancer, Wang et al. [46] synthesized different novel makaluvamine analogues and evaluated their activities in breast cancer. In particular FBA-TPQ analogue induced apoptosis in different breast cancer cell lines and inhibited MCF-7 xenograft tumour growth in vivo.

Manzamine A is an alkaloid isolated from sponges of the genus *Haliclona* sp., *Xestospongia* sp. and *Pellina* sp., which demonstrated different pharmacological properties such as anti-inflammatory, antimicrobial and anticancer effects [47]. It showed pro-apoptotic effect in human pancreatic adenocarcinoma cells and human colorectal cancer cells [48]. In particular, tetraethylbenzimi-dazolylcarbocyanine iodide (JC-1) staining, which is used for the evaluation of the membrane integrity, showed that manzamine A induces depolarization of mitochondrial membrane potential and consequent release of cytochrome c which in turn triggers the downstream apoptotic cascades [49].

Monanchocidin A is an alkaloid extracted from the marine sponge *Monanchorapulchra* [50] which has promising anti-cancer properties. In particular, its cytotoxic activity has been demonstrated in both leukemic and ovarian human cancer cells (THP-1 and Hela respectively) [50]. Moreover, monanchocidin A also exhibits anti-migratory activity in different bladder cancer cell lines and induces autophagy and lysosomal membrane permeabilization [51].

Renieramycin M is a tetrahydroisoquinolinequinone alkaloid isolated from the blue sponge *Xestospongia* that revealed an encouraging cytotoxic activity in colon, lung and breast cancer cell lines in the range of nanomolar concentrations [52]. Renieramycin M cytotoxic effect is mainly related to apoptosis as determined by flow citometry analysis. Interestingly, renieramycin M is also able to stimulate anoikis in H460 lung cancer cells as confirmed by the XTT assay. Moreover, it also induces inhibition of anchorage-independent growth, invasiveness and migration. Thus, these data suggest that renieramycin M also possesses potential anti-metastasis properties [53].

Nortopsentins A–C are alkaloids isolated from the sponge *Spongosoritesruetzleri* [54]. These molecules showed in vitro cytotoxicity against P388 leukemia cells. However, due to the very small amount of this substances isolated from the sponge, different nortopsentins synthetic approaches have been proposed [55]. These semi-syntethic compounds showed antiproliferative effect on HCT-116 colorectal cancer cells at low micromolar concentrations. In addition, they induced apoptosis and cell cycle arrest also by inducing mitochondrial dysfunction [56].

#### 3.1.2. Terpenoids Isolated from Marine Sponges

10-Acetylirciformonin B is a furanoterpenoid that was extracted and purified from marine sponge *Ircinia sp.* This molecule showed cytotoxic activity against HL-60 human leukemia cells in a dose- and time-dependent manner [57]. Its cytotoxic effect was associated to apoptosis as demonstrated by flow cytometry analysis using Annexin V and propidium iodide double staining. The pro-apoptotic effect involves induction of DNA strand breakages and phosphorylation of H2A.X and p-CHK2 that are markers evaluated during nuclear DNA damage [58]. In addition, treatment of HL 60 cells with 10-acetylirciformonin B for 24 h increased the activation of caspases-3, -8 and -9 as well as the cleavage of PARP.

Heteronemin is a sesterterpene extracted from the sponge *Hyrtios*, that showed different biological activity and cytotoxic effect on several cancer cell lines including leukemia, colon, breast, cervical and renal cancer in the range of nanomolar concentrations [59]. Heteronemin regulates both extrinsic and intrinsic apoptotic pathways in chronic myeloid leukemia K562 and human renal cell carcinoma A498 cells by inhibiting MAPK and AKT signallings which are involved in inflammation and apoptosis [59,60]. More recently Lee et al. [61] reported that heteronemin exerts a pro-apoptotic effect in LNcap prostate cancer cells. In addition, they also reported that heteronemin induced also autophagy in LNcap cells as confirmed by the expression of LC3-II, a soluble protein involved in autophagic activity [62].

Stellettins A and B are triterpenoids isolated from the sponge *Stellettatmuis* collected from China. Stellettin A was significantly toxic to P388 leukemic cells at micromolar range [63], whereas stellettin B showed an antiproliferative and pro-apoptotic effect in SF295 gliobastoma cell lines [64]. Furthermore, Liu et al. [65] showed that both stellettin A and stellettin B induced oxidative stress and apoptosis in HL-60 human leukemia and LNCaP prostate cancer cell lines confirmed by the activation of caspase-8, the reduction of Bcl-2 protein expression and the increase of FasL.

Smenospongine is a sesquiterpene extracted from the Indonesian sponge *Dactylospongiaelegans* that has been reported to induce apoptosis and G1 arrest in HL60 and U937 leukemia cells in a dose-dependent manner [66]. Moreover Tang et al. [67] also reported a pro-apoptotic effect of smenospongine in MCF-7 human breast cancer by increasing the phosphorylation level of p38 and AMPKα that have been reported to inhibit cell growth and induce apoptosis.

#### 3.1.3. Macrolides Isolated from Marine Sponges

Peloruside A is a macrolide isolated from the marine sponge *Mycale hentscheli* that demonstrated cytotoxic and apoptotic effect on human (HL-60) and mouse (32D-ras) myeloid leukemic cells, as well as non-transformed 32D cells [68]. Another similar compound is laulimalide isolated from the sponge *Cacospongia*. It also showed a pro-apoptotic effect in MDA-MB-435 human breast cancer cell lines by inducing mitotic arrest and activation of the caspase pathways [69]. Moreover, it has been reported that both peruloside and laulimalide show a mechanism of action similar to that of paclitaxel and other taxol drugs binding to β-tubulin and inducing polymerization and stabilization [70].

Salarins are a class of nitrogenous macrolides isolated from the marine sponge *Fascaplysinopsis* in Madagascar. Among the different salarins, salarin C is the most potent inhibitor of cell proliferation as demonstrated in vitro on K562 leukemia cells. Salarin C induced apoptosis and cell cycle arrest in a dose- and time-dependent manner. This effect was also confirmed by the activation of caspase-3 and -9 and the consequent PARP cleavage. In addition, it has also been demonstrated that salarin C induces mitochondrial permeability alteration supporting cythocrome c release [71].

Tedanolides are a small family of macrolides which includes tedanolide, 13-deoxytedanolide and candidaspongiolide. The latter is isolated from the marine sponge *Candidaspongia* and has been reported to promote apoptosis in human U251 glioma and HCT116 colon carcinoma cells by triggering caspase-3 and -12 activation [72]. In addition, it has been also demonstrated that candidaspongiolide inhibits proliferation of human melanoma cells in a selective manner compared to breast and lung cancer cell lines [73]. However, the underlying reasons for the selectivity of candidaspongiolide in melanoma cell lines have not been determined and are currently being investigated.

#### 3.1.4. Other Molecules Isolated from Marine Sponges

Agelasine B is a toxin isolated from the marine sponge *Agelasclathrodes*. This toxin demonstrated cytotoxic effect on two different human breast cancer cell lines (MCF-7 and SKBr3) and on PC3 human prostate cancer cells. In particular, agelasine B releases Ca^2+^ from the endoplasmic reticulum that is correlated to induced DNA fragmentation and apoptosis [74]. This data has been also further confirmed by the activation of caspase-8 and the reduction of the anti-apoptotic protein Bcl-2 [75].

Dideoxypetrosynol A is a polyacetilene extracted from the *Petrosia sponge*. It has been widely studied for its pro-apoptotic effect on SK-MEL-2 human melanoma cancer cells and U937 human leukemia cells. In particular, dideoxypetrosynol A-induced chromatin condensation and formation of apoptotic bodies in SK-MEL-2 melanoma cells as demonstrated by morphological analysis with DAPI staining. Moreover, the addition of dideoxypetrosynol A to SK-MEL-2 cells increased accumulation of the sub-G1 phase in a dose-dependent manner quantified by flow cytometry of fixed nuclei [76]. In U937 leukemia cells, dideoxypetrosynol A also showed cell cycle arrest demonstrated by evaluating the expression levels of the cell cycle regulating factors such as cyclin D1, cyclin E, Cdk2, Cdk4 and Cdk6 [77].

Jasplakinolide, also called jaspamide, is a macrocyclic peptide isolated from the marine sponges *Jaspis johnstoni* and *splendens* [78]. Jasplakinolide induces apoptosis in Jurkat T cells through the activation of the executioner caspase-3 and the increase of BCL-2 pro-apoptotic proteins family such as BAX [79]. Moreover, studies focused on the murine IL-2-dependent T cell line CTLL-20 showed that the pro-apoptotic role of jasplakinolide is related to the stabilization of polymerized actin which has been reported to be involved in the transduction of apoptotic signal [80].

Psammaplysene A is a bromotyrosine derivative isolated from the sponge *Psammaplysilla*. It showed an interesting pro-apoptotic activity against two different type of human endometrial cancer cell lines Ishikawa and ECC-1. In particular, Psammaplysene A pro-apoptotic effect is linked to the nuclear export inhibition of FOXO1 transcription factor, which in turn triggers apoptosis by activating the pro-apoptotic protein Bim or inducing the tumour necrosis factor-related apoptosis-inducing ligand (TRAIL)-mediated apoptosis pathway [81]. This result has been also confirmed by using silenced FOXO1 ECC-1 cells and overexpressing FOXO1 Ikasawa cells in which have been evaluated the cleavage of PARP substrate and its correlation with FOXO1 [82]. Moreover, psammaplysene A also showed a cooperative effect with cisplatin in reduction of cell viability and induction of apoptosis in HT29 colon cancer cells [83] and in lung cancer cells [84].

### 3.2. Marine Algae

Among marine organisms, marine algae, which include microalgae and macroalgae (or seaweeds), constitute a major source, after sponges, of natural compounds with diverse biological activities. Microalgae constitute more than 90% of the oceanic biomass and they are single-cell photosynthetic organisms distinguished in prokaryote (cyanobacteria) and eukaryote [85]. Actually, more than 10,000 species are known, and many of them are edible and rich in essential nutrients such as protein, fibre, vitamins, minerals and polyunsaturated fatty acids. In addition, they have also revealed to be a prolific source of secondary metabolites (polysaccharides, phycobilins, polysaccharides, sterols, tocopherols, terpenes, polyphenols and phycocyanins) with numerous bioactive properties such as antioxidants, anticancer, anti-inflammatory, antihypertensive, anti-hyperlipidaemia, anticoagulant, immunomodulatory, neuroprotective, antiviral and antimicrobial [86]. Traditionally, seaweeds have been utilized in cuisine and medicine in East Asian countries, where there has been observed a lower incidence of chronic diseases, such as hyperlipidaemia, coronary heart disease, diabetes and cancer, compared to Western countries [87]. Finally, marine algae have also the advantages of a faster cultivation, processing, and harvesting cycle, as well as the ability to be cultured on waste materials, which enhance drug cost effectiveness and biopotential. A list of marine algae compounds with pro-apoptotic effects is reported in Table 3. 

#### 3.2.1. Cyanobacteria

Cyanobacterium (marine blue-green algae or Cyanophyceae) are slow-growing photosynthetic prokaryotes, which are responsible for producing atmospheric oxygen as well as numerous bioactive secondary metabolites with several pharmacological properties [88]. Most of these metabolites have a structure type common to a subset of natural products (i.e., rifamycin, lovastatin, vancomycin, bleomycin) utilized as therapeutic agents in some human diseases, including inflammation, bacterial infections and cancer. In fact, they are produced by a biosynthetic pathway that involve either the non-ribosomal polypeptide (NRP) synthase and the mixed polyketide-NRP synthase, resulting in chemically different structures such as linear peptides, cyclic peptides, linear lipopeptides, depsipeptides, cyclicdepsipeptides, fatty acid amides, swinholides, glicomacrolides or macrolactones [89]. The cytotoxic effects of marine cyanobacteria compounds on human tumour cell lines are the most studied, and several compounds have emerged as templates for the development of new anticancer drugs. Among the mechanisms implicated in their cytotoxicity, one of the most studied is the activation of the apoptotic process. Some compounds were found to induce the activation of caspase-3, while others were found to induce cell cycle arrest or mitochondrial dysfunctions or alterations in BCL-2 protein family, important regulators of apoptosis in cancer [90]. Secondary marine cyanobacterial metabolites mainly derived from *Oscillatoriales* orders followed by *Nostocales* and *Chroococcales*. In particular, more than 300 nitrogen-containing metabolites have been isolated from two marine filamentous cyanobacterial genera: *Lyngbya* and *Symploca* [91].

Apratoxins, a class of potent cytotoxic cyclic depsipeptides originally isolated from various strains of *Lyngbya* sp. from Guam, Palau, and Papua New Guinea, exhibited high potency against various cancer cells. Apratoxin A, the parental compound isolated from *Lyngbya majuscule*, was shown to induce pronounced G1-phase cell cycle arrest and apoptosis in osteosarcoma U2OS cells by activation of caspase-3 and -7 [92]. Apratoxin E, isolated from *Lyngbya bouilloni*, also displayed strong cytotoxicity against several cancer cell lines derived from colon, cervix and bone [93].

New cyclic depsipeptides, lagunamide A, lagunamide B and lagunamide C were recently also isolated from *Lyngbya majuscula*, from the western lagoon of Pulau. Lagunamides metabolites displayed potent cytotoxic activity when tested against a panel of cancer cell lines such as P388, A549, PC3, HCT8 and SK-OV3It, which might occur via induction of mitochondrial-mediated apoptosis [94,95].

Hectochlorin and lyngbyabellins are structurally-related lipopeptide and cyclic depsipeptides isolated from the genus *Lyngbya*, which are described to inhibit cell cycle proliferation in a human Burkitt lymphoma cell line by inducing an arrest in G_2_/M phase [96,97]. Dolastatins are cytotoxic peptides that were initially isolated from the sea hare *Dolabella auricularia* and later found to be produced by marine cyanobacterial strains *Symploca* [98]. Dolastatin 10, the cyanobacterial metabolite, was found to induce G_2_/M arrest and the activation of the apoptotic mitochondrial “intrinsic” pathway in human lung carcinoma cells [99]. Dolastatin 10 and its synthetic derivative Soblidotin have been used in trials for the treatment of sarcoma, leukemia, lymphoma, liver cancer and kidney cancer (https://clinicaltrials.gov/ct2). Similar to dolastatin 10, symplostatin 1, another metabolite isolated from the genus *Symploca*, exhibited a great efficacy in inhibiting a variety of cancer cell proliferation. Symplostatin 1 caused the formation of abnormal mitotic spindles and accumulation of cells in metaphase resulting in a G_2_/M cell cycle arrest. Moreover, symplostatin 1 initiated the phosphorylation of Bcl-2, formation of micronuclei and activation of caspase-3, indicating induction of apoptosis [100].

Kanamienamide, an enamide with an enol ether 11-membered lipopeptide, isolated from *Moorea bouillonii*, in Kagoshima, exhibited growth-inhibitory activity in HeLa cells and caused apoptosis-like cell death in HeLa cells trough caspases cascade activation [101].

Spirulinales are filamentous cyanobacteria with trichomes regularly spirally coiled, with parietal thylakoids, lacking heterocytes. Previously belonging to the Synechococco phycidae family, now they are considered a new order following whole genome sequencing. However, “*Spirulina platensis*”, commercially important for its phylogenetic and cytological criteria has been classified in the genus Arthrospira (Oscillatoriales) [102]. *Spirulina platensis* is widely used as a dietary supplement because of its high nutritional value; it is rich in proteins, essential fatty acid, vitamins and mineral but is also potent in antioxidants including spirulans (sulphated polysaccharides) and phycobiliproteins (C-phycocyanin and allophycocianin). Spirulina has been demonstrated to have hypolipidemic, hypoglycemic and antihypertensive properties. Recently, *S. platensis*-derived bioactive substances have also been investigated for their anti-cancer functions. In particular, c-phycocyanin, a light-harvesting biliprotein, seems to be implicated in most of the biological effects of spirulina. Indeed, several studies have shown that phycocyanin plays anti-proliferative and pro-apoptotic effects on different cancer cell types such as lung cancer [103], liver cancer [104], colon cancer [105], breast cancer [106,107] and leukemia [108] both in vitro and in vivo, without side effects on normal tissue. C-phycocyanin can affect cancer cell cycle proliferation by inducing cell cycle arrest in G0/G1 phase and by activating the apoptotic pathway in several ways. For example, in MDA-MB-231 breast cancer cells, the antitumor effect of c-phycocyanin was mediated by induction of apoptosis trough upregulation of Fas protein levels of and cleavage of caspase-3, while downregulation of Bcl-2 protein was observed [107]. In K562 leukemia cells, the c-phycocyanin pro-apoptotic effect was mediated by release of cytochrome c from mitochondria into the cytosol instead [108]. In addition, MAPK, PI3K/Akt/mTOR and NF-κB pathways are also involved in phycocyanin-induced apoptosis [103,107]. Interestingly, when phycocyanin was combined with chemotherapeutic drugs, their efficacy was improved resulting in reduction of the dose, and consequently, minimizing the adverse effects. It is reported that diverse drugs may synergize with phycocyanin to kill human cancer. The combination of piroxicam (the traditional non-steroidal anti-inflammatory drug) and phycocyanin was more than 70% effective to inhibit colon cancer development in rat than single-use drugs [109]. Moreover, Bing Li [110] found that an all-trans-retinoic acid (ATRA) and c-phycocyanin combination treatment of HeLa cells could significantly upregulate the expression of caspase-3 while downregulating the anti-apoptotic protein Bcl-2. In addition, c-phycocyanin and betaine combination decreased A549 cell viability up to 60%, and might effectively inhibit tumour growth in rats [111]. C-phycocyanin could be also combined with He-Ne laser for the treatment of tumours, acting as photosensitizer. Thus, when tumour cells (HepG2, MCF-7, MDA-MB-231) were irradiated, the pre-treatment with phycocyanin induced mitochondrial membrane potential loss, increased reactive oxygen species (ROS), released cytochrome c and activated caspase-3, all effects leading cells to apoptosis [106,112,113]. These studies demonstrated the great therapeutic potential of phycocyanin in cancer therapy.

Two potent bioactive metabolites belonging to the *Nostocales* order are represented by calothrixin A and cryptophycin 52. The calothrixins are quinone-based natural products isolated from *Calothrix* cyanobacteria that have shown potent antiproliferative effects against several cancer cell lines. Interestingly, calothrixin A has been found to display potent antiproliferative effects against leukemia cells. Beyond a cell cycle block, calothrixin A is described to induce an oxidative stress in Jurkat human T cells that as a consequence foments DNA fragmentation leading to apoptosis [114]. Calothrixin B has shown antiproliferative activity against the HCT-116 colon cancer cell line instead [115]. Cryptophycin 52 is a naturally macrocyclic depsipeptide isolated from the marine cyanobacteria *Nostoc* spp. [116]. Cryptophycin 52 is an antimicrotubules agent with potent antiproliferative and cytotoxic activity against a broad spectrum of cultured human tumour cell lines, including several multidrug-resistant lines as well as murine tumours model [117]. The cytotoxic effects of cryptophycin 52 appear to be mediated, at least in part via apoptosis. In particular, in several prostate cancer cell lines including PC-3, LNCaP and DU-145, cryptophycin 52 induced apoptosis via proteolytic processing and activation of caspase-3 and caspase-1 and cleavage of the caspase substrate PARP. In addition, cell death was also associated with phosphorylation of Bcl-2 and Bcl-x(L) proteins and upregulation of p53 protein and the p53-regulated gene *p21* [118]. Also cryptophycin 1 is described to induce apoptosis in a human ovarian carcinoma cell line, initiating the caspases cascade through caspase-3 activation [119]. Finally, both cryptophycins were found to induce DNA fragmentation [118,119].

Natural oxadiazinenocuolin A (NoA) with a unique 1,2,3-oxadiazine heterocycle (N–N–O) structure was identified in three independent cyanobacterial strains of genera *Nostoc*, *Nodularia*, and *Anabaena.* It has been reported that NoA-induced cell death is clearly consistent with apoptosis as demonstrated by blebbing, caspase activation and PARP-1 cleavage in HeLa cells [120].

To this end, among the members with anti-proliferative activity belonging to Chroococcales order, there is coibamide A, a depsipeptide founded in a Panamanian *Leptolyngbya* sp. This compound was evaluated in the National Cancer Institute’s NCI’s panel of 60 cancer cell lines, and it exhibited significant activities against human breast cancer (MDA-MB-231), human melanoma (LOX IMVI), human leukemia (HL-60) and human glioblastoma (SNB-75)cells at nanomolar concentrations [121]. Coibamide A was also described as capable to cause the arrest of cell cycle progression in G_1_ phase in melanoma-like MDA-MB-435 cells and in U87-MG and SF-295 glioblastoma cells [121,122]. A deeper analysis of the mechanisms of action of coibamide A showed caspase-3 activation in dying cells [123].

#### 3.2.2. Chlorophytes

Chlorophyta is a group of green photosynthetic microalgae with biological and pharmacological properties important for human health. They have a long history of use as food source for human consumption, aquaculture and animal feed, as well as for other commercial purposes such as colouring agents, cosmetics and others [124]. Many species of green algae are able to produce several secondary metabolites when they are exposed to stress or sub-optimal conditions linked to nutrient deprivation, light intensity, temperature, salinity and pH which are health-promoting agents. Some of them have also shown promising anticancer activity. Out of 174 strains from 24 genera of green microalgae screened, 10 strains from the species *Desmococcus olivaeceus*, *Chlorella* and *Scenedesmus* showed anticancer activity [125]. The best-known example is *Chlorella* *vulgaris* which has been widely used for centuries as a food supplement for its health benefits and its high nutritive value due to the adequate contents in protein, omega-3 polyunsaturated fatty acids, polysaccharides, vitamins and minerals. Clinical trials have suggested that supplementation with *C. vulgaris* can ameliorate hyperlipidemia and hyperglycemia, and protect against oxidative stress, cancer and chronic obstructive pulmonary disease [126]. Concerning the anticancer properties several studies have been demonstrated that *Chlorella* extracts exhibit cytotoxic effects in various human cancer cell lines. The anti-cancer effects of *C. Vulgaris* derivatives have been related to their ability to modulate apoptosis signalling pathways. In fact, *C. vulgaris* has been shown to inhibit cell proliferation and to induce apoptosis cascades in the hepatoma cell line HepG2 [127], in human lung carcinoma A549 and NCI-H460 cells [128] and in rat models of liver cancer [129]. The mitochondrial signalling pathway was involved in *C. vulgaris*-induced apoptosis of HepG2 cells with increased expression of pro-apoptotic proteins p53, Bax and caspase-9/3 and decreased expression of Bcl-2 protein [127]. In the same way the chemopreventive role of *C. vulgaris* in hepatocarcinogenesis-induced rats was mediated by the mitochondria-dependent apoptotic pathway through caspase-8 activation. Other species of green algae belonging to the genus *Chlorella* have also demonstrated anticancer effects: *C. sorokiniana* has shown to induce apoptosis in non-small cell lung cancer (NSCLC) cells through the activation of the mitochondrial-mediated apoptotic pathway associated to downregulation of BCL-2 anti-apoptotic family, which resulted in a marked reduction of tumour growth in mice [130]; *C. pyrenoidosa* induced antineoplastic effects in experimental breast cancer model, instead [131]. Green algae contain also many active pigments such as carotenoids, which are normally used as colouring agents. Generally, carotenoids in plants play important roles in the photosynthetic process, such as light-absorption and quenching of excess energy [132]. Interestingly, several carotenoid extracts from marine *Chlorella* have demonstrated antioxidant and protective activity on human cells. In particular, carotenoid extracts from *C. ellipsoidea* (violaxanthin, xanthophylls, antheraxanthin and zeaxanthin) and from *C. vulgaris* (lutein) have been shown to inhibit human colon cancer HCT116 cell proliferation. Between the two species, pro-apoptotic effect of *C. ellipsoidea* extract was almost 2.5 times stronger than that of the *C. vulgaris* extract [133]. Among carotenoids, algal lycopene isolated from *C. Marina* also showed very significant anti-proliferative and pro-apoptotic effect in human prostate cancer cell lines [134]. *Dunaliella salina* is a unicellular halophilic green microalga with potent antioxidant activity and plays an important role in reducing the risk of several ROS-associated diseases, such as cancer. *Dunaliella salina* is known to be a rich source of β-carotene. In particular, under stress conditions, its production increases and as a consequence, the antioxidant and cytotoxic activities of *D. Salina* results enhanced [135,136]. The extracts from *D. salina* are widely used as nutrition supplement especially in China, Japan and Taiwan, particularly as natural antimicrobials and antioxidants. The cytotoxic effect of *D. salina* has been studied in different cancer cell lines [137,138]. An ethanol extract of *D. salina*, that is higher in β-carotene content, was able to significantly inhibit carcinogenesis in a murine model of squamous cell cancer by enhancing cell immunity [139]. Further, carotenes enriched extracts of *D. salina* showed significant antioxidant and cytotoxic effects in breast cancer cell lines (MCF-7) [140]. Sheu et al. reported that the anticancer effect of *D. Salina* was apoptotis-mediated. They demonstrated that ethanol extract of *D. salina* induced the activation of the extrinsic apoptotic FasL/FasR pathway in A549 lung cancer cells via upregulation of p53. Consequently, the apoptosis-inducing effect of p53 resulted in caspase-8 activation and increased expression of the pro-apoptotic protein Bax [141]. These finding were also confirmed in an in vivo model of mammary carcinogenesis. In this study, female tumour bearing-rats treated whit *D. salina* lyophilized powder showed up to 83.4% reduction of tumour volume. This chemopreventive effect was obtained through the suppression of cell proliferation and the induction of apoptosis [142]. *Haematococcuspluvialis* is also a rich source of carotenoid. Astaxanthin (3,3′-dihydroxy-β, β′-carotene-4,4′-dione) a xanthophyll carotenoid has demonstrated antioxidants properties and efficient promotion of apoptosis in prostate cancer cell lines by inhibiting NF-kappa B [143]. Siphonaxanthin is a specific keto-carotenoid of siphonaceous green algae. It represents the major carotenoid in the peripheral light-harvesting complexes (LHCs) of photosystem II and helps to absorb green and blue-green light under water [144]. In edible green algae such as *Codium fragile*, *Cauler palentillifera* and *Umbraulva japonica*, siphonaxanthin content is approximately 0.03–0.1% of the dry weight. Interestingly, siphonaxanthin has been reported to induce apoptosis in human leukemia cells (HL-60). The induction of apoptosis was associated with the decreased expression of Bcl-2, and the subsequently increased activation of caspase-3. In addition, siphonaxanthin upregulated the expression of GADD45α and of the DR5 [145].

*C. fragile* is also an important source of clerosterol, which is a cholesterol derivative [146]. The cytotoxic effects and mechanism of action of clerosterol were investigated in A2058 human melanoma cells. Cells treated with clerosterol showed chromatin condensation and DNA fragmentation, which are typical morphologic changes associated with apoptosis. Indeed, clerosterol induced apoptosis in A2058 cell via the mitochondria-mediated pathway, which was associated with the modulation of Bcl-2 and Bax expression, and an increase in the levels of cleaved caspases-9 and -3 [147].

Recently, polysaccharides from marine organisms have also garnered attention because of their potential anti-tumour, immune-modulatory and antioxidant effects [148]. The greatest antitumor properties have been reported for ulvans, which are polysaccharides from green microalga from the genus *Ulva*. Polysaccharides isolated from *Ulva lactuca*, a marine alga widely distributed at Egyptian sea shores were reported to have anti-proliferative effects on different types of cancer in vitro and in vivo. Kaeffer et al., were the first to show that ulvan sulphated polysaccharide inhibits proliferation of tumoural colon carcinoma cell lines (HCT-29, HCT-116, and Caco-2) [149]. Later, other studies revealed the potential anticancer effects of those ulvans also on tumoural hepatocellular carcinoma cell lines (HepG2) and in Ehrlich ascites carcinoma (EAC)-bearing mice [150]. Finally, ulvan polysaccharides isolated from *U. lactuca* have shown potent antiproliferative and cytotoxic effect against breast carcinoma cell line in vitro and potential chemopreventive effects against breast carcinogenesis in vivo [151]. The anticarcinogenic and ameliorative effects of these polysaccharides have been demonstrated to be mediated through the enhancement of an antioxidant defence system and induction of apoptosis, which might be associated with the increased expression of p53 and decreased expression of Bcl-2 proteins [150,151]. Sulphated polysaccharides isolated from *Ulva pertusa* showed little cytotoxicity against gastric adenocarcinoma (AGS) cells while demonstrating strong immunomodulatory activity by stimulating Raw 264.7 cells to produce high amounts of nitric oxide and various cytokines [152]. Polysaccharides obtained from *Ulva fasciata*, also known as sea lettuce, are reported to be naturally high in Se. Selenium plays an important role in many physiological processes and it has been reported to have potent chemopreventive activities [153]. Selenium-polysaccharide, which contain about 44.4 μg/g of Se, has been demonstrated to gain greater anti-tumour effect than native polysaccharides. It has shown potent anti-proliferative activity against human lung cancer A549 cells, leading to apoptotic mitochondrial-mediated pathway by activating caspase-9 and down-regulating the expression of BCL-2 family proteins [154]. A sulphated polysaccharide, extracted from *Ulva intestinalis*, is found to have anticancer activity on human hepatoma HepG2 cell line. It induces apoptosis on HepG2 cells involving a caspases-mediated mitochondrial signalling pathway, which is associated with changes in mitochondrial membrane potential, release of cytochrome c to the cytosol, activation of caspase-9 and -3, as well as decrease of Bcl-2 expression and increase of Bax expression [155]. *Capsosiphon fulvescens* was shown to inhibit the AGS cell proliferation and induced apoptosis by inhibiting insulin-like growth factor-I receptor (IGF-IR) signalling and the PI3K/Akt pathway, which resulted in caspase-3 activation and decrease of Bcl-2 expression levels [156].

A glycoprotein (GLP) with a molecular mass of 48 kDa, extracted and purified from *Codium decorticatum*, was able to inhibit cell viability by inducing apoptosis in human MDA-MB-231 breast cancer cells. Apoptosis was associated with alterations in the mitochondrial membrane potential, which led to mitochondrial membrane permeabilization, cytochrome c release, and activation of caspases-3 and -9 [157].

#### 3.2.3. Rhodophyta

Rhodophyta, also known as red algae, have been documented as a source of natural nutraceuticals and pharmaceuticals for many years. Recent studies showed that sulphated polysaccharides isolated from red seaweed possess various therapeutic and biological feature such as anti-oxidants, anti-proliferative, anti-tumour, anti-viral and anti-coagulants [158,159].

Genus *Laurencia* are one of the most studied marine organisms among the red algae. They are found on the inter-tidal rocks of the warm sea waters throughout the world. There are almost 421 species actually known. It has been provend that the genus *Laurencia* is a rich source of structurally unique secondary metabolites, characterized by a relatively high degree of halogenation and core terpene structures. The halogenated metabolites from *Laurencia* possess several biological activities, such as antifeedant, anthelmintic, antimalarial, antifouling, antimicrobial and cytotoxicity [160,161]. Two halogenated sesquiterpenes (obtusol and elatol) isolated from *L. dendroidea* showed a great antiproliferative activity on colorectal adenocarcinoma cells (Colo-205) obtained through the induction of apoptosis. Elatol activity proceeded trough the activation of caspases-2, -4, -6 and -8, on the other hand obtusol activity involved only caspase-6 activation [162]. Similar effects were induced also by the analogue elatol isolated from algae *L. microcladia*, which inhibited tumour growth in B16F10 melanoma-bearing mice. The anti-tumour properties of elatol were, at least in part, related to cell cycle delay in the G(1)/S transition coupled with the simultaneous induction of apoptosis. In particular, the apoptotic pathway was associated with an increase of p53 expression and caspase-9 cleavage and to a decreased of Bcl-xl protein [163]. Recently, the inhibition of the DEAD-box RNA helicase eIF4A1 has been identified as a new possible mechanism of action for the anticancer activity of elatol [164]. Laurinterol is a marine sesquiterpene extracted from *L. Okamurai*. Beside its well-known antimicrobial activity, laurinterol has demonstrated to be able to induce apoptosis through a p53-dependent pathway in melanoma cells (B16F1). Anticancer activity of laurinterol against melanoma cells was determined by DNA fragmentation and caspase activation [165]. The marine polyether triterpenoid dehydrothyrsiferol isolated from *L. viridis* induced apoptosis on in hormone insensitive breast cancer cells by estrogen-dependent and independent pathways [166]. Mertensene, a halogenated monoterpene from the red alga *Pterocladiella capillacea*, inhibited human colorectal adenocarcinoma cell (HT29 and LS174) proliferation. In HT29 cancer cells, mertensene triggered a caspase-dependent apoptosis characterized by caspase-3 activation and PARP cleavage. In addition it induced G2/M cell cycle arrest associated with a reduction of the cellular level of cyclin-dependent kinases (CDK) 2 and 4. Moreover, the antiproliferative effects seems correlated to the modulation of MAPK ERK1/2, Akt and NF-κB pathways, the major regulators of cell survival, proliferation and metabolism [167]. A new class of diterpene-benzoate macrolides, namely bromophycolides, were isolated from *Callophycus serratus*. The first identified, bromophycolide A, demonstrated moderate in vitro cytotoxic potency on A2780 human ovarian cells, consequent to the arrest at the G1 phase of the cell cycle and to a simultaneous induction of apoptosis [168]. Later, other bromophycolides (C-I) were obtained from *C. serratus* with cytotoxic activity against a wide range of cancer cells. Among them, the antiproliferative effect of Bromophycolide D was the most significant [169].

Sulphated polysaccharides extracted from red seaweeds have gained enormous attention over the last decades for their multiple chemical structures and biological activities, particularly for their potential anti-cancer properties [170]. Carrageenans are a small chemical group of linear sulphated polysaccharides, which represent the main composite constituent compounds of cell walls in a large group of edible red algae. Carrageenans are divided into three categories (κ, I or λ) according to their degree of sulfation and water solubility. λ-carrageenan, which had the highest degree of sulfation, exhibited an efficient anti-proliferative activity in the human breast cancer cells MDA-MB-231 via the promotion of caspases-dependent apoptosis and with the contribution of the pro-apoptotic protein Bax. The altered Bax/Bcl-2 ratio led to the disruption of mitochondrial transmembrane potential with the consequent release of cytochrome c triggering the formation of the APAF-1/caspase-9 complex and subsequently inducing the active form of caspase-3 [171]. Similar results were obtained also with degraded ι-carrageenan, which inhibited cell proliferation in Caco-2 and HepG2 cancer cell lines via apoptosis induction [172]. An extracted sulphated carrageenan (ECS), predominantly consisting of ι-carrageenan obtained from the red alga *Laurencia papillosa*, was characterized for its potential cytotoxic activity against the MDA-MB-231 cancer cell line. This study demonstrated that the algal ESC has anti-proliferative properties that lead to apoptosis. Indeed, ECS induced the extrinsic pathway of apoptosis in MDA-MB-231 cells via the recruitment of caspase-3, caspase-8 and caspase-9, the re-modulation of the Bax/Bcl-2 ratio and DNA damage [173]. Further, λ- and ι-carrageenan isolated from *L. papillosa* was shown to significantly inhibit MCF-7 breast cancer cell viability by inducing apoptosis-mediated cell death [174].

The next good candidate chemotherapeutic agent, acting through the induction of apoptosis is 2,3,6-tribromo-4,5-dihydroxybenzyl methyl ether (TDB), isolated from *Symphyocladia latiuscula*, which has been shown to induce apoptosis in the MCF-7 cells via caspase-3 activation with PARP cleavage and Bcl-2 downregulation. Treatment with TBD also caused a marked increase in the level of p21WAF1/CIP1 protein in a p53-dependent manner and DNA fragmentation [175].

Eucheuma serra agglutinin (ESA) is a lectin derived from a marine red alga *Eucheuma serra* inducing tumour cell death by apoptosis. It has been proposed that the antitumour activity of ESA might be related directly to specific intermolecular interactions with unique sugar chains rich in mannose localized on the tumour cell surface. Such specific binding between ESA and cancer cells led to apoptosis activation. Indeed, several studies reported that ESA induced cell death against several cancer cell lines, such as colon cancer (Colo201 and Colo26), cervix cancer (HeLa) and osteosarcoma (OST and LM8). DNA ladder detection and the induction of caspase-3 activity confirmed apoptosis induction by ESA in cancer cells [176,177]. Moreover, an intravenous injection of ESA in the tail of Colon26 tumour-bearing mice significantly inhibited tumour growth by inducing apoptotic cell death, without any serious side-effects for the animals [178]. Furthermore, the administration of PEGylated Span 80 vesicles with ESA encapsulated, is expected to express more effective antitumor activity against cancer cells, increasing the potential of ESA as new effective anti-tumour drug [179].

#### 3.2.4. Phaeophyta

Phaeophyta (brown algae) represent a large group of photoautotrophic marine macro-algae, which include about 265 genera with more than 1500 species. They produce a plenty of secondary metabolites, which have been demonstrated to be potent antibiotic, antifungal, antiviral, or anticancer agents. Among them, brown-algal polysaccharides and carotenoids received considerable attention because of their proven health effects such as antibacterial, antioxidant, anti-inflammatory, anticoagulant and antitumor effects. Utilization of these compounds as an ingredient in some dietary supplement products has been very common in the East Asian countries, particularly in Japan, China and Korea. In addition brown-algal polysaccharides are present in large amounts in algal biomass and are easy to isolate permitting their use as functional food supplements or nutraceuticals [180].

Fucoidans and laminarans represent the most abundant polysaccharides in the cell wall of various species of brown algae, and the principle carbohydrate reserve. Fucoidans are a complex series of sulphated polysaccharide that exists mainly in the cell wall matrix of brown seaweeds such as mozuku, kombu, limumoui, bladderwrack and wakame and are the main components of the sticky substance. The basic structure of fucoidan consists of fucose monosaccharide units with attached sulphate groups. More complex molecular structures of fucoidans strongly depend on the species and other factors such as harvesting time and habitat conditions [181]. Recently, a number of studies have obtained interesting results regarding the anticancer properties of fucoidans both in vitro and in vivo, in different types of cancers. Clinical trials of patients with breast, cervical, renal, and hepatic carcinomas showed a significant improvement in tumour regression among patients who received an alternative medicine treatment regimen based on fucoidan administration [182]. Fucoidan isolated from *Fucus vesiculosus* and *Undaria pinnatifida* are the most studied for the comprehension of their chemical and biological properties. Various mechanisms have been postulated for the anticancer activity of fucoidans, such as induction of cell cycle arrest, apoptosis and immune system activation [183]. In particular, it has been demonstrated that in a wide variety of cancers, such as hematopoietic, lung, breast, prostate and colon cancers, fucoidans mediated cell death through induction of apoptosis [184,185,186,187,188,189,190]. Numerous studies have investigated the underlying pathway of fucoidan-induced apoptosis. The extrinsic pathway, which is initiated by activation of death receptors leading to cleavage of caspase-8, was induced by fucoidan in the MCF-7 breast cancer cell line [191]. In contrast, Zhang et al. found that fucoidan from *Cladosiphono kamuranus* induced apoptosis in MCF-7 through a caspase-independent pathway involving mitochondrial permeabilization, activation of Bcl-2 family proteins, and release of cytochrome c. Thus, they concluded that fucoidan performed its activity through alteration of mitochondrial function. Fucoidan from *F. vesiculosus* activated caspases via both the death receptor-mediated and mitochondria-mediated apoptotic pathways in human colon cancer cells [185]. Similarly, in HL-60 cells fucoidan treatment induced activation of caspases-8, -9 and -3, the cleavage of Bid, and changed the mitochondrial membrane permeability. In addition MEKK1, MEK1, ERK1/2, and JNK, resulted in important mediators in fucoidan-induced apoptosis of human leukemic cells [190]. Treatment of MDA-MB231 breast cancer cells with low molecular weight fucoidan resulted in a significant decrease in anti-apoptotic proteins Bcl-2, Bcl-xl and Mcl-1 associated with the activation of caspases, mitochondrial dysfunction and alteration of Ca^2+^ homeostasis [192]. Compared to other sulphated polysaccharides, the fucoidans extracted from the sporophylls of the brown seaweed *Undaria pinnatifida* showed higher sulphate and L-fucose content, and exhibited a broader range of bioactivities [193]. On human hepatocellular carcinoma (SMMC-7721) cells, Yang et al. demonstrate that the mechanism responsible for fucoidan-induced apoptosis was mediated by ROS. They showed that the increasing ROS production in fucoidan-exposed cells induced mitochondrial oxidative damage, mitochondrial membrane potential depolarization and a release of cytochrome c, with final activation of caspase-9 and caspase-3 [194]. Recently, the same ROS-dependent apoptotic pathway was also reported in human bladder cancer cells (5637HBCC) after treatment with fucoidan. According to the authors, the anticancer effect of fucoidan was related to: (i) increased Bax/Bcl-2 expression ratio; (ii) dissipation of the mitochondrial membrane potential; and (iii) production of intracellular ROS [195]. The pro-apoptotic effects of fucoidan from *U. Pinnatifida* were also examined on A549 human lung carcinoma cells. In this case, fucoidan was shown to induce caspase-9 activation following upstream downregulation of p38 MAPK and PI3K/Akt pathway [184]. Boo et al. (2013) found that fucoidan isolated from *U. Pinnatifida* induced apoptotic cell death in human prostate cancer cells (PC-3) through the activation of both intrinsic and extrinsic molecular signalling pathways. Thus, fucoidan treatment led to upregulation of DR5 and cleavage of caspase-8, which are critical in the extrinsic pathway; fucoidan also led to the downregulation of Bcl-2, upregulation of Bax, and activation of caspase-9, which are essential in the intrinsic pathway. Moreover, their results demonstrated that induction of apoptosis following fucoidan addition to in PC-3 cells was associated with downregulation of PI3K/Akt and Wnt/β-catenin signalling pathways [187]. It is interesting to note that some studies have shown that fucoidan and laminaran may enhance the effects of anticancer drugs. For example, the combination of fucoidan with arsenic trioxide (ATO) and ATRA, two well-known synthetic anticancer drugs, significantly reduced the development of the tumour mass in mice bearing NB4 cells (acute promyelocytic leukemia) [196]. Combining fucoidan with standard chemotherapeutic agents resulted in increased apoptosis in cancer cell while reducing chemotherapy-related side effects [192,197]. A recent study showed that fucoidan in combined therapy with cisplatin exerts a greater inhibition of tumour volume in a mouse model of lung cancer. Interesting, an increasing number of clinical results suggested that by combining cisplatin and fucoidan important anticancer effects were obtained in lung cancer patients [198]. Actually, the effect of fucoidan plus platinum-based chemotherapy on quality of life in subjects with NSCLC (stage III and stage IV) (NCT03130829) is under examination by the FDA (https://clinicaltrials.gov/ct2). Innovative fucoidan-coated copper sulphide nanoparticles (F-CuS) have been designed for chemo-photothermal therapy. Fucoidan-coated copper sulphide nanoparticles may potentiate cancer treatment by combining the fucoidan-mediated apoptosis and therapeutic effect of laser irradiation. In addition, using near-infrared light-responsive nanoparticles, the intracellular delivery of fucoidan in cancer cells results also improved. Indeed, this combination effectively eliminated multiple tumours in live mouse models [199].

Laminarans are brown algal polysaccharides mainly composed of β-glucan (β1–3, β1–6-glucan). Beside the structural characteristics, laminarans also exhibit some biological activities of other glucans such as immune-stimulating and anticancer effects, as well as antibacterial activity. Generally, laminarans are present in the cell wall of brown algae (mainly in Laminaria/Saccharina, and to a lesser extent in Ascophyllum and Fucus species) in an amount of about 35% of seaweed dry weight, which can vary according to seaweed species, harvesting season, habitat and extraction method [180]. The antitumor effect of laminarans has been described in different cancer types. Park et al. reported that laminaran obtained from *Laminaria japonica* inhibited human colon cancer HT-29 cell growth by decreasing cell proliferation and inducing apoptosis. The proposed mechanism of cell death involved Fas-mediated apoptosis because laminaran was found to increase the expression of Fas and FADD protein levels, which in turn induced the activation of caspases cascade. Further results demonstrated that laminaran downregulated IGF-IR, whose overexpression plays a part in cancer cell proliferation and protects cancer cells against apoptosis [200]. In a successive study, laminaran was also shown to induce a dose-dependent sub-G1 and G2-M phase cell cycle arrest, followed by apoptosis in highly proliferative colon cancer cells. The authors found that laminaran antiproliferative effect was dependent on the inhibition of ErbB signalling pathway-related proteins [201]. Similar finding were also confirmed by Ji and Ji, who investigated the underlying mechanisms of laminaran-induced apoptosis in human colon cancer (LoVo) cells. Their results revealed that laminaran induces apoptosis through both extrinsic and intrinsic pathways. Indeed, they observed upregulation of DR-4, DR-5, TRAIL and FADD, which activated caspases-8, -3, -6, and -7 as well as the activation of the mitochondrial pathway, which led to the release of cytocrom c and the activation of caspase-9 and -3 [202,203].

Fucoxanthin is one of the most abundant carotenoids of some brown algae, such as wakame (*Undaria pinnatifida*) and kombu (*Laminaria japonica*), which have a long history of use in the diets of Asian cultures and in traditional Chinese medicine. In humans, dietary fucoxanthin is mainly metabolized in the gastrointestinal tract by digestive enzymes to fucoxanthinol, the deacetylated form of fucoxanthin, which is absorbed into intestinal cells. Then, circulating fucoxanthinol is further converted to amarouciaxanthin A in the liver. Fucoxanthinol is considered to be the active form of fucoxanthin and it had a stronger inhibitory effect on the viability of human cancer cells [204]. Fucoxanthins are known to have many health benefits such as anti-inflammatory, anti-obesity, anti-diabetes, hepato-protective and cardiovascular-protective activities [205]. Moreover, accumulating evidence has shown that fucoxanthins and its metabolite fucoxanthinol exert their anti-proliferative and cancer preventive action in several cancer cells and mouse models. The mechanisms of action underlying this anticancer effect mainly relay on the induction of cell-cycle arrest and apoptosis in several tumour cell lines [206,207]. In many cases fucoxanthin was shown to induce cell-cycle arrest during the G_0_/G_1_ phase in a dose- and time-dependent manner. The induction of cell-cycle arrest was accompanied to a reduction of the kinase activity of the cyclin D/CDK4 complex. Concomitantly, a caspase-dependent apoptosis was induced, together with downregulation in some apoptosis-related proteins such as Bcl2, XIAP and cellular inhibitor of apoptosis protein 2 (CIAP2). Additionally, the effects of fucoxanthin and fucoxanthinol might be mediated through the inactivation of transcription factor NF-κB or AP-1 [208,209,210,211]. Several studies report that fucoxanthin induced DNA fragmentation in human prostate cancer cells PC-3, DU145 and in human colon cancer cells Caco-2 [212,213] or induced mitochondrial-dependent apoptosis in human promyelocytic leukemia HL-60 [214]. Moreover, it has been reported that fucoxanthin induced caspase-dependent apoptosis following ROS generation and Bcl-XL reduction in HL-60 cells [215]. The same pro-apoptotic effect caspase-mediated was also observed in human bladder cancer cells, in human cervical cancer cells HeLa [216]; in human breast cancer cells MCF-7 and in estrogen-resistant cells MDA-MB-231 [204]. In gastric cancer SGC-7901 cells, fucoxanthin induced autophagy, which in turn promoted apoptosis with caspase-3 cleavage and Bcl-2 downregulation [217]. There is some evidence showing that fucoxanthin could be used to potentiate the action of conventional anticancer drugs. Liu et al. observed that fucoxanthin improved chemotherapeutic efficacy of cisplatin by enhancing cisplatin-induced apoptosis in HepG2 cells.

Fucoxanthin combined with cisplatin also attenuated the expression of DNA repair genes, such as *ERCC* (excision repair 1), and thymidine phosphorylase, which are responsible of platinum resistance in cancer cells [218]. Temozolomide is an alkylating agent widely used in the therapy of malignant gliomas. However, the main disadvantage related to this drug is the development of resistance. There is evidence that the combination of fucoxanthin with temozolomide could be used to potentiate the action of anticancer drugs in glioblastoma treatment and to overcome the resistance issue. Promising data have been raised also for the treatment of colon cancer through the combination of 5-fluorouracil (5-FU) and fucoxanthin. In fact, it has been demonstrated that the marine compound enhances the cytotoxic effect of 5-FU in colon cancer cells, whereas no marked cytotoxicity was observed in normal colon cell lines (CCD-18Co) [219]. Recently, the effects of TRAIL and fucoxanthin combination treatment has been investigated. TRAIL, a member of the TNF superfamily, can selectively trigger apoptosis in tumour cells and has the ability to circumvent the chemoresistance of conventional therapeutics. Jin et al. [220] found that fucoxanthin enhanced TRAIL sensitivity in TRAIL-resistant human cervical cancer SiHa cells, which resulted in increased cell apoptosis. Other metabolites from *Phaeophyta* algae have also been described for their anticancer activity.

Bis (2,3-dibromo-4,5-dihydroxybenzyl) ether (BDDE) is a marine bromophenol compound isolated from the marine algae *Leathesia nana* exhibiting broad and potent cytotoxicity against several cancer cell lines (HeLa, HCT-116, HCT-8, SMMC-7721, A549, and K562 cells). BDDE arrests cell cycle in S phase and apoptosis on K562 cells by a mitochondrial mediated pathway [221]. HFGP from *Hizikia fusiformis* [86] and LJGP from *Laminaria japonica* [222] induced apoptosis on HepG2 and HT-29 cells, which was mediated by the Fas signalling pathway. Diphlorethohydroxycarmalol, isolated from *Eiseniabicyclis*, induced apoptosis on HL60 cells through the accumulation of sub-G1 cell population along with nuclear condensation, the reduction of Bcl-2 expression and the depletion of mitochondrial potential [223].

## 4. Conclusions

The inhibition of apoptosis is known as one of the hallmarks of cancer. The ability of cancer cells to escape from programmed cell death contributes to tumour growth, promotes tumour cell spread to distal organs and confers resistance to cytotoxic anticancer agents. Therefore, bioactive compounds with the ability to inhibit cancer cell proliferation by activating apoptotic pathways can be employed as potential chemotherapeutic drugs. Marine environments offer an abundant source of new bioactive molecules with unique structural features and a wide range of biological activities, such as antiviral, anti-bacterial, anti-fungal, antiparasitic, anti-oxidant, anti-inflammatory and anti-tumour. Among all marine organisms, sponges and algae represent the richest source of natural marine compounds which have received considerable attention because of their anticancer activity. Several studies have shown that both sponge- and algae-derived compounds inhibit tumour growth and progression through different mechanisms, such as cell-cycle arrest, anti-inflammatory activity, apoptosis, induction of ER stress and inhibition of angiogenesis. In particular, here we reviewed the underlying cellular mechanisms by which these different compounds induce apoptosis of tumour cells. Apoptosis occurs via two principal pathways: the extrinsic (cytoplasmic) pathway whereby death receptors trigger the apoptosis, or the intrinsic (mitochondrial) pathway in which changes in mitochondrial membrane potential lead to cytochrome C release and death signal activation. The two apoptotic pathways converge on caspase-3 activation which is the main effector of apoptosis. The evidence reported in this review highlights the great value of many marine derivatives to promote cell death by activating one or both apoptotic pathways in cancer cells. Moreover, some studies have shown that some of the compounds isolated from sponges or algae may enhance the chemotherapeutic efficacy of commercial anticancer drugs. Therefore, these marine natural products might represent promising sources for new anticancer drug development; however, their utilization as co-adjuvants in therapeutics should also be evaluated.

## Figures and Tables

**Table 1:** Marine compounds isolated from sponges and algae with IC_50_ value.

Compound	Chemical Structure	IC50
**Fascaplysin**	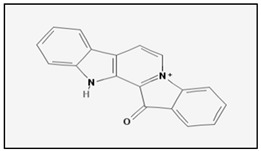	SCLC cell lines 0.96 µM NSCLC cell lines 1.15 µM Breast and ovarian cancer cell lines 0.89 µM
**Monanchocidin A**	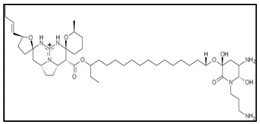	HCT 116 4.5 µM DLD-1 and HT-29 >10 µM
**10-Acetylirciformonin B**	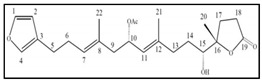	HL-60 1.7 g/mL
**Heteronemin**	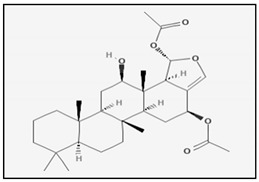	ACHN 3.54 µM A498 1.57 µM
**Laulimalide**	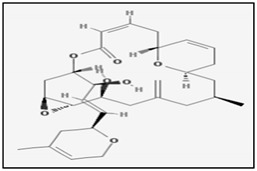	MDA-MB-435 5.74 nM SK-OV-3 11.53 SKVLB 1210
**C-phycocyanin**	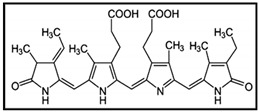	S-HepG2 40 µM R-HepG2 50 µM K562 50 µM MDA-MB-231 194.4 µg/mL
**Elatol**	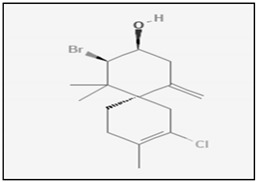	COLO-205 2.5 µg/mL L929 1.1 µM B16F10 10.1 µM
**Fucoidan**	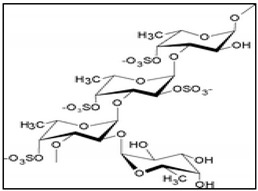	WiDr 0.244 mg/mL MCF-7 0.173 mg/mL
**BDDE**	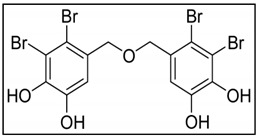	K562 13.9 µg/mL

**Table 2:** Pro-apoptotic compounds isolated from marine sponges.

Compound	Mitochondrial Pathway	Death Receptor Pathway	Caspases Activation	Other Pathways
**Alkaloids**				
Aaptamine				NFκB/AP-1
Isoaptamine			3 and 7	
Crambescidin 800			3	
Fascaplysin			3	AKT/AMPK
Lamellarin D	✔			
Makulavamines			3, 8 and 9	
Manzamine A	✔		9	
Monanchocidin A			3 and 7	
Renieramycin M			3 and 7	
Nortopsentins A-C	✔			
**Terpenoids**				
10-Acetylirciformonin B			3, 8 and 9	
Heteronemin	✔	✔	3, 8, 9 and 10	
Stellettins A and B		✔	8	
Smenospongine				p38/AMPK
**Macrolides**				
Peloruside A			3 and 7	
Salarins	✔		3 and 9	
Tedanolides			3 and 12	
**Others**				
Agelasine B	✔		8	Bcl-2
Dideoxypetrosynol A	✔		3 and 9	BAX/Bcl-2
Jasplakinolide			3	BAX/Bcl-2
Psammaplysene A		✔		FOXO1

**Table 3:** Pro-apoptotic compounds isolated from marine algae.

Compound	Mitochondrial Pathway	Death Receptor Pathway	Caspases	Other Pathways
**Cyanobacteria**				
Apotoxin A			3 and 7	
Lagunamide A-C	✔			
Dolastatin 10	✔			
Symplostatin 1			3	Bcl-2
Kanamienamide			3 and 7	
C-phycocyanin	✔	✔	3	NF-κB/MAPK/AKT
Cryptophycin 52			3 and 1	p53/Bcl-2
Cryptophycin 1			3	
NoA			3 and 7	
Coibamide A			3	
**Chlorophytes**				
*C. vulgaris extracts*	✔		3, 8 and 9	p53/Bcl-2/BAX
*C. sorokiniana extracts*	✔			Bcl-2
*D. salina extracts*		✔	8	p53/BAX
Astaxanthin				NF-κB
Siphonaxanthin			3	Bcl-2
Clerosterol	✔		3 and 9	Bcl-2/BAX
Ulvans	✔		3 and 9	p53/Bcl-2/BAX
GLP	✔		3 and 9	
**Rhodophyta**				
Obtusol			6	
Elatol			2, 4, 6, 8 and 9	p53/Bcl-XL/eIF4A1
Laurinterol			3	p53
Mertensene			3	MAPK/AKT/NF-κB
λ and ϊ-carrageenans	✔		3 and 9	Bcl-2/BAX
ECS			3, 8 and 9	Bcl-2/BAX
TDB			3	Bcl-2/p53
ESA			3	
**Phaeophyta**				
*F. vesciculous* Fucoidans	✔		3, 8 and 9	Bcl-2/Bcl-XL/MAPK
*U. Pinnatifida* Fucoidans	✔	✔	3, 8 and 9	p38/AKT/ROS Bax/Bcl-2/β-cat
Laminarans	✔	✔	3, 6, 7, 8 and 9	IGF-IR
Fucoxanthin	✔		3 and 9	NF-κB/AP-1/ROS/Bcl-XL
BDDE	✔		3 and 9	Bax/Bcl-2/ROS
LJGP		✔	3, 8 and 9	p27/Bcl-2
Diphlorethohydroxycarmalol	✔			Bcl-2
